# The Effect of Osseodensification on Implant Stability and Marginal Bone Levels: A Randomized Control Clinical Trial

**DOI:** 10.1002/cre2.70126

**Published:** 2025-04-10

**Authors:** Ioanna Politi, Bahman Honari, Lewis Winning, Ioannis Polyzois

**Affiliations:** ^1^ Department of Restorative Dentistry and Periodontology Dublin Dental University Hospital, University of Dublin, Trinity College Dublin Ireland; ^2^ Department of Biostatistics Dublin Dental University Hospital, University of Dublin, Trinity College Dublin Ireland

**Keywords:** bone levels, implant stability, osseodensification

## Abstract

**Objectives:**

To compare the implant stability quotient values (ISQ) of implants placed using implant‐specific drills (CD) and osseodensification drills (OD) at three different time points and to determine the effect of both drilling techniques on marginal bone levels.

**Material and Methods:**

38 subjects were recruited and ISQ values and radiographic marginal bone levels were recorded after surgery (T1), and also at 3 (T2) and 4–5 months (T3). Clinical and radiographic marginal bone levels were also recorded.

**Results:**

At T1, mean ISQ values ranged from 65.5 to 81 for the CD group and 29 to 80 for the OD group. For the CD group, ISQ values were 72.20 ± 2.6 (95% CI) at T1, 75.0 ± 2.0 at T2, and 74.8 ± 2.3 at T3. The corresponding ISQ values for the OD group were 68.1 ± 5.6, 71.9 ± 1.6, and 72.2 ± 2.4, respectively. Implants placed using CD drills showed greater stability at 3 months but not at placement or at 4–5 months. No statistically significant differences were identified regarding marginal bone levels between the two groups.

**Conclusions:**

There was a notable increase in implant stability over time for both treatment modalities. At T2, implants inserted into osteotomies made with standard drills exhibited significantly greater stability compared to those placed using OD drills. However, the clinical relevance of this difference is questionable, as it was not observed at T3. Marginal bone levels were comparable for both groups over all time points.

**Trial Registration:**

ClinicalTrials.gov identifier: NCT05376020

## 1 Introduction

Osseodensification (OD) is an innovative technique that has gained attention for its potential to improve implant primary stability compared to conventional drilling (CD). Unlike traditional drills, OD utilizes specially designed Densah burs that can rotate in both clockwise and counter‐clockwise directions. This counter‐clockwise motion allows the burs to compact bone and auto‐graft it in an outward, expanding direction towards the osteotomy walls. The result is a denser bone structure surrounding the lateral and apical regions of the osteotomy site (Hema and Ashwin 2018).

The theory behind this system is that the lateral compaction of autogenous bone during OD leads to the generation of residual strain, which causes a spring‐back effect. This spring‐back exerts gentle compressive forces against the implant, fostering a mechanical interlock between this and the bone, thereby enhancing primary implant stability (Trisi et al. 2016). Furthermore, compacted bone fragments generated during OD are thought to act as nucleating agents, potentially accelerating osteogenesis and new bone formation, thus improving implant stability throughout the healing process (Huwais and Meyer 2017).

Concerns have been raised regarding the potential for excessive strain around the peri‐implant tissues when using the OD technique (Halldin et al. 2011). Whilst bone tissue is capable of withstanding certain levels of strain due to its elastic properties, excessive strain may lead to micro‐cracks in the surrounding bone, which can result in extensive interfacial bone remodeling during healing. This may, in turn, contribute to marginal bone loss (MBL) (Halldin et al. 2011; Stocchero et al. 2016; Bonfante et al. 2019). Furthermore, in addition to micro‐cracks, excessive implant insertion torque may lead to compression necrosis. This occurs when the blood supply to the bone is compromised, impairing cellular viability and osseointegration (Nascimento et al. 2024).

Preclinical research on the effects of OD has produced mixed outcomes (Huwais and Meyer 2017; Almutairi et al. 2018; Delgado‐Ruiz et al. 2020). Moreover, clinical studies assessing OD's impact on primary implant stability remain limited. Only three controlled trials have directly compared implant stability between OD and CD, yielding contrasting results (Ibrahim et al. 2020; Bergamo et al. 2021; Sultana et al. 2020). Most other clinical studies are either retrospective or prospective without control groups, and only one has evaluated OD's effect on marginal bone levels (Koutouzis et al. 2019; de Carvalho Formiga et al. 2022a).

Therefore, the aim of this study was to compare the implant stability quotient (ISQ) values of implants placed using implant‐specific drills (CD) and osseodensification drills (OD) at three distinct time points. Additionally, the study aimed to assess the impact of both drilling techniques on MBL, while also measuring soft tissue thickness and insertion torque at implant placement to control for their potential influence on MBL outcomes.

## 2 Materials and Methods

### 2.1 Study Design

**Figure 1:**
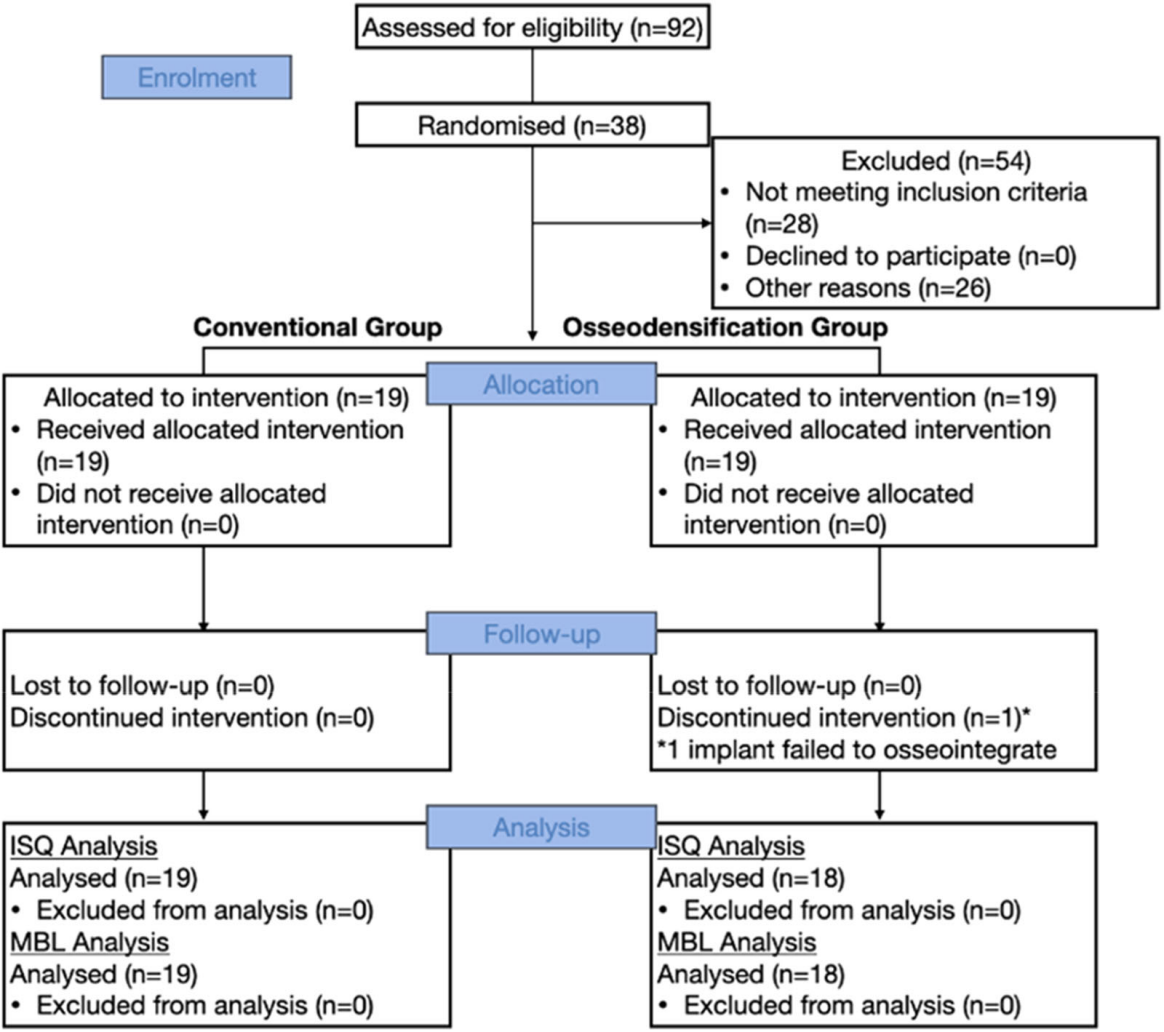
Consolidated Standards of Reporting Trials (CONSORT) 2010 Flow Diagram demonstrating how participants were assessed for eligibility and randomized following inclusion into conventional (control) or osseodensification (intervention) groups.

The study was designed as a parallel arm, randomized control clinical trial on a cohort of Dublin Dental University Hospital patients who were having dental implants placed in the maxilla only. The procedures were conducted in conformity with the 2013 Helsinki declaration and CONSORT guidelines (Figure 1). An application for ethical approval was submitted to St James' Hospital research ethics committee (JREC) and subsequently approved in April 2022 (Project ID: 0612). It was also registered on ClinicalTrials.gov (NCT05376020) in September 2022 and before recruitment commenced. A detailed description of the protocol was given to each patient included in the study, and a signed inform consent was acquired in order for them to be included. A single operator (IP) performed all implant placements and a single experienced examiner (IP*), blinded to the surgical drill protocol utilized, performed all clinical and radiographic assessments.

An a priori power calculation was performed based upon the available data in the literature on ISQ values which was considered as the primary outcome of this study (Bergamo et al. 2021; Sultana et al. 2020). To achieve 80% power, a sample of 19 subjects was required in each treatment group. Therefore, a total of 38 participants were enrolled in this study.

### 2.2 Patient Selection

General inclusion criteria were as follows: Need for implants in the Maxilla, 18 years old or over, capacity to provide informed consent, willing to comply with study appointment schedule, sufficient bone volume present for implant placement without the need for bone graft/augmentation.

Exclusion criteria were as follows: Plaque score > 20%, bleeding score > 20%, heavy smokers (> 10 cigarettes per day), untreated periodontal disease, uncontrolled systemic disease, use of systemic medications with an expected impact on bone healing, need for mandibular implants and previously grafted sites.

Only one implant was placed in each patient. If a single patient was receiving more than one dental implant in the maxilla and there were numerous implant sites that qualified for entry into the research, only the most posterior implant site was selected for the study.

At the time of surgery, each patient selected a numbered, sealed envelope with the treatment group allocation. Each envelope corresponded to a number on a computer‐generated randomization list created before enrollment of the study participants. Depending on what was revealed when the envelope was opened the patient had her/his implant placed using CD or OD.

### 2.3 Surgical Procedure

All the implants placed in this study were ZimVie T3 With DCD Non‐Platform Switched Certain Tapered Implants (BNST) of varying length (8.5–13 mm) or width (3.25, 4.1, or 5 mm).

Preoperatively all the patients were instructed to rinse with 0.2% chlorhexidine solution for 1 min. After local anesthesia was given (2% lidocaine with 1:80,000 adrenaline), full thickness mucoperiosteal flaps were raised buccally and palatally and implant osteotomies were performed under copious saline irrigation. The osteotomies were performed in accordance with the recommended drilling protocols for each of the techniques.

For the conventional group, standard drilling at a speed of 1200–1500 rpm as recommended by ZimVie was followed. Initially, the osteotomy site was marked with a round rose‐head bur. Subsequently, a 2 mm Initial Twist Drill was used, followed by the appropriate Quad Shaping Drills depending on the diameter of the implant to be placed.

For the intervention group, Densah burs were used for OD following the protocol recommended by Versah. As per the protocol, the drilling speed was kept in the range between 800 and 1500 rpm. Initially, a pilot drill was used in a clockwise direction to the desired length. Different diameters of Densah burs were used subsequently in counter‐clockwise direction depending on the diameter of the implant to be placed and the quality of the bone. As the Densah burs didn't exactly match the shape of the BNST ZimVie implants/drills (Figure 4) the protocol for the coronal part of the osteotomy had to be modified to allow for good fitting of the implant into the osteotomy prepared. The coronal 2 mm of the osteotomies prepared for a BNST 3.25, 4.1, and 5 mm implants were prepared in Osseodensifyng mode with a 4, a 4.5, and a 5.5 mm Densah drill, respectively.

Implants were inserted with the motor handpiece set at 40 Ncm without irrigation. Following recording of the implant stability and marginal bone levels, the cover screws were placed on the implants. The flaps were repositioned and sutured, leaving the implants submerged.

Postoperative instructions were given to the patients, including a soft diet and rinsing with 0.2% chlorhexidine mouthwash twice daily for 2 weeks. The patients were given a prescription for Amoxicillin 500 mg, one tablet every 8 h for 5 days. The patients were reviewed in 2 weeks.

### 2.4 Measurement Outcomes

Measurement outcomes and time period are summarized in Table 1. Three months following implant placement the patients attended for the second‐stage implant surgery. Local anesthesia was administered and full thickness mucoperiosteal flaps were raised buccally and palatally over the sites where the implants were placed. Following recording of the implant stability and marginal bone levels, healing abutments were placed on the implants and the flaps were repositioned and sutured. The patients were subsequently reviewed at 6 weeks post second‐stage surgery when they attended for an appointment to have the implants restored. Final recordings were taken at that appointment (Table 1).

**Table 1:** Table of measurement outcomes.

	Baseline (T1)	3 Months (T2)	4–5 Months (T3)
Insertion Torque	✓		
Soft tissue thickness	✓		
Marginal bone levels (Clinical)	✓	✓	
MRFA (Osstell)	✓	✓	✓
Marginal bone levels (Radiographs)	✓	✓	✓

The primary outcome was Implant Stability which was assessed by magnetic resonance frequency analysis (MRFA) using the Osstell ISQ device (Osstell AB, Gothenburg, Sweden). At each of these visits, a SmartPeg was mounted to the platform of the implant using the screw channel and was hand‐tightened as per the manufacturer's instructions by IP*. Each SmartPeg was single‐use and specific for the implant placed. Mesio‐distal and bucco‐lingual measurements were taken twice on each implant.

Clinical measurements of the marginal bone levels were taken at the time of implant placement and at second‐stage surgery (Table 1). At the time of implant restoration, marginal bone levels could not be recorded clinically as no flaps were raised.

The marginal bone levels were recorded clinically at four sites (mid‐buccally, mid‐palatally, mid‐mesially, mid‐distally) with a Hu‐Friedy UNC periodontal probe (Hu‐Friedy, Chicago, IL, USA). The same probe was used to measure soft tissue thickness.

**Figure 2:**
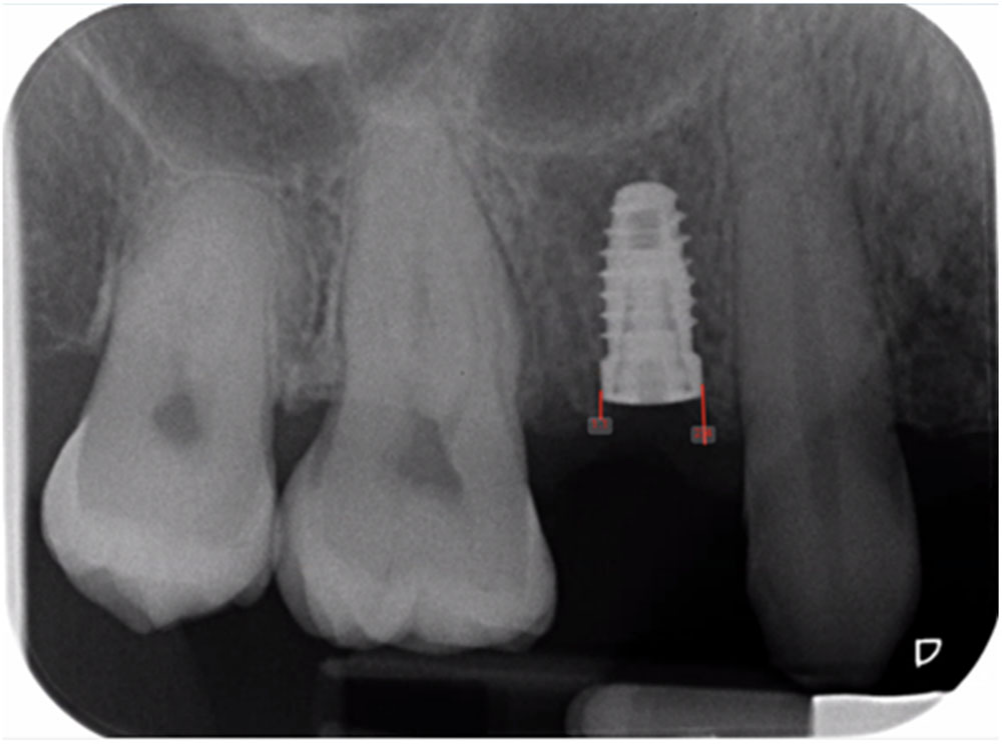
Radiographic measurement of marginal bone levels. Mesial and distal measurements were taken at each implant from the platform to the first bone contact.

Intra‐oral peri‐apical radiographs of the surgical site were taken at implant placement, at second‐stage surgery and at implant restoration to assess the radiographic marginal bone levels. The radiographs were taken using long‐cone paralleling technique and phosphor plates, which were developed using a digital scanner. One examiner (IP*) assessed the radiographs using Planmeca Romexis Pro software. Radiographs were pre‐calibrated to a known dimension (film size), and mesial and distal measurements were taken at each implant from the platform to the first bone contact (Figure 2).

**Figure 3:**
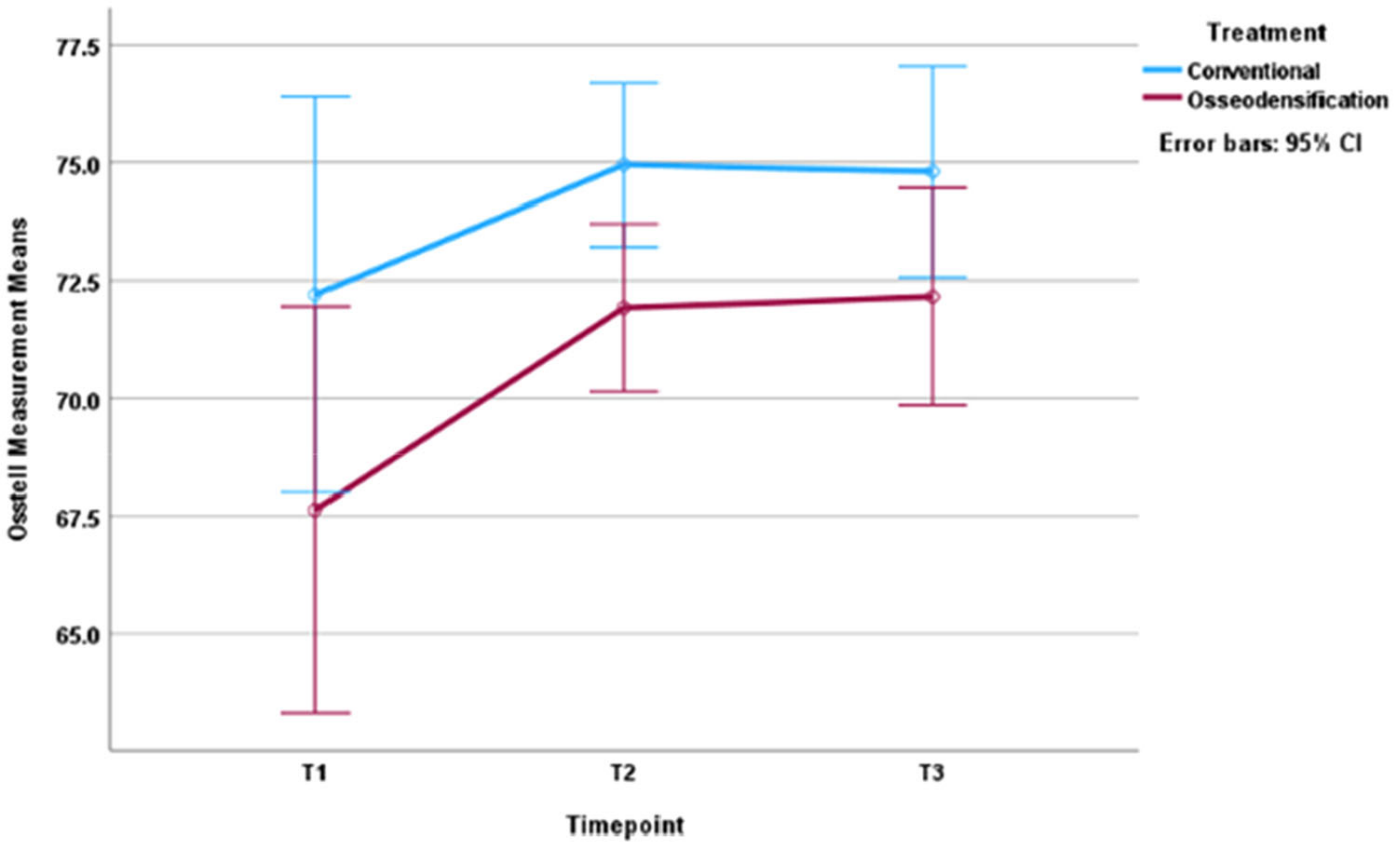
Graphical representation of ISQ values for conventional and osseodensification groups.

Insertion torque (IT) was recorded using the implant motor (W&H Implantmed classic). Implants were inserted with the motor handpiece set at 40 Ncm. If the implants were fully seated before the motor handpiece stopped, the IT was recorded as < 40 Ncm. If the motor handpiece stopped before the implants were fully seated, then the IT was recorded as > 40 Ncm and installation of these implants was completed with a manual surgical torque wrench indicator (Figure 3).

### 2.5 Statistical Analysis

Descriptive statistics including mean values and 95% confidence intervals (CI) were calculated for each variable.

Analyses of the ISQ values at different time points and the changes between time points were shown to be normally distributed using the Shapiro–Wilk test. Repeated measures mixed ANOVA was used for the analysis of the difference in mean ISQ values at different time points (within‐subject analysis), as well as at different levels of factors: treatment type, implant diameter, implant length, implant site, and insertion torque (between‐subject analysis). For the within‐subject analysis, Mauchly's test of sphericity was performed to ensure the sphericity value is met (*p* > 0.05). When the sphericity assumption was not met, the effect of the within‐subject factor was assessed using the Greenhouse‐Geisser statistic. For the between‐subject analysis, Levene test was performed and showed homogeneity of variance across different groups of the between‐subject factors. The Shapiro–Wilk test of normality was performed for the clinical and radiographic marginal bone level values at different time points. The data were not normally distributed, and therefore Mann–Whitney or Kruskal–Wallis tests were used.

The level of statistical significance was set at *p* < 0.05. Analyses were performed using SPSS version 29 (IBM Corp., Armonk, NY, USA).

## 3 Results

Thirty‐eight patients were included in the study, with no dropouts recorded. Twenty‐three (60.5%) females and 15 (29.5%) males with a mean age of 42.7 years (±16.3 years [SD], range 20–70 years). Of the patients enrolled, 27 (71%) were non‐smokers, 9 (23.7%) were ex‐smokers, and 2 (5.3%) were smokers reporting to smoke 5 or less cigarettes per week. There were 19 patients in the control group and 19 patients in the intervention group.

Twelve implants were placed in hypodontia patients, and hence, a permanent tooth was not present before implant placement. Two implants were placed as immediate implants but without a need for simultaneous grafting. The remaining 24 implants were placed either as early Type 1 (*n* = 2), early Type 2 (*n* = 3), or late (*n* = 19) (Gallucci et al. 2018). None of the teeth replaced with a dental implant were extracted due to periodontal problems. One tooth was lost due to trauma and the remaining teeth were lost due to restorative or endodontic complications. Four implants were placed in the incisor area, 7 in the canine area, 23 in the premolar area, and 4 in the molar area. Out of the 38 implants, 35 implants were placed in sites with thick (≥ 2 mm) ST and 3 were placed in sites with thin (< 2 mm) ST.

Eleven implants had a length of 8.5 mm, 23 implants had a length of 10 mm, 3 implants had a length of 11.5 mm length, and 1 implant was 13 mm long. The diameter of implants also varied. Nine implants were 3.25 mm wide, 24 were 4 mm, and 5 were 5 mm wide.

IT was high (> 40 Ncm) for 13 out of the 38 implants and low (< 40 Ncm) for the remaining 25 implants.

One implant from the OD group failed to osseointegrate and had to be removed before second‐stage implant surgery as it presented clinically with a sinus tract, therefore it was automatically removed from the analysis of outcome change, and outcome change percentage between time points.

### 3.1 Implant Stability Quotient Values

The ISQ values for the control and intervention group at each time point are shown in Table 2. At the time of implant placement, the ISQ values ranged between 65.5 and 81 for the conventional group and 29–80 for the OD group. For the control group, the ISQ values were 72.20 ± 2.6 (95% CI) at T1, 75.0 ± 2.0 at T2, and 74.8 ± 2.3 at T3. The corresponding ISQ values for the intervention group were 68.09 ± 5.6, 71.9 ± 1.6, and 72.2 ± 2.4, respectively (Figure 3).

**Table 2:** Implant stability quotient values.

Implant stability quotient values – All cases												
Factor	Factor level (*n*)	At time of implant placement (Time 1)			At time of second‐stage surgery (Time 2)			At time of implant restotation (Time 3)			*p* (within) individual	*p* (within)
		Mean (95% CI)		*p*	Mean (95% CI)		*p*	Mean (95% CI)		*p*		
Treatment	Conventional (19)	72.20	(69.62, 74.77)	0.133	74.95	(72.92, 76.98)	0.019	74.80	(72.46, 77.14)	0.104	T1–T2: 0.036	0.018
	Osseodensification (18)	68.09	(62.51, 73.68)		71.92	(70.35, 73.48)		72.15	(69.77, 74.53)		NS	(T1–T2): 0.014, (T1–T3): 0.025
Site	Premolars and molars (27)	69.94	(65.74, 74.13)	0.958	73.66	(72.02, 75.29)	0.650	73.81	(71.97, 75.66)	0.550	T1–T2: 0.047	—
	Incisors and canines (10)	70.66	(67.97, 73.35)		72.98	(70.32, 75.63)		72.70	(68.54, 76.86)		NS	—
Torque	< 40 (12)	74.31	(71.95, 76.66)	0.056	73.81	(70.85, 76.78)	0.725	74.46	(70.98, 77.93)	0.428	NS	—
	> 40 (25)	67.98	(63.71, 72.25)		73.31	(71.78, 74.84)		73.06	(71.11, 75.01)		T1–T2: 0.007, T1–T3: 0.022	—
Implant diameter	3.25 mm (8)	69.59	(66.75, 72.43)	0.883	71.78	(68.30, 75.26)	0.133	71.16	(67.87, 74.44)	0.243	NS	—
	4.00 mm (23)	70.76	(66.44, 75.08)		71.38	(71.77, 74.99)		73.82	(71.59, 76.04)		NS	—
	5.00 mm (6)	68.42	(57.21, 79.63)		76.08	(71.80, 80.36)		75.50	(70.43, 80.57)		NS	—
Implant length	8.5 mm (13)	66.71	(58.81, 74.62)	0.288	73.56	(70.78, 76.34)	0.792	73.52	(70.44, 76.59)	0.655	T1–T2: 0.039	—
	10.0 mm (20)	72.24	(70.24, 74.24)		73.26	(71.43, 75.10)		73.41	(71.03, 75.79)		NS	—
	11.5 mm (3)	71.42	(59.21, 83.62)		75.33	(70.16, 80.50)		75.83	(65.27, 86.40)		NS	—
	13.0 mm (1)	72.50	N/A		71.00	N/A		68.50	N/A		N/A	—

Overall, and for all implants in the study included, ISQ values changed significantly over time, the difference was significant between T1 and T2 (*p* = 0.014), and between T1 and T3 (*p* = 0.025). When looking at each group individually, ISQ values changed significantly only for the control group from T1 to T2 (*p* = 0.036). The control group showed higher mean ISQ values at all three time points when compared to the OD group, but the difference between the groups was statistically significant only at T2; (*p* = 0.019) (Table 2).

The percentage increase in ISQ values when compared to baseline (T1) was calculated for T2 and T3. Mean ISQ values increased by 4.2% ± 3.7% at T2% and 4.0% ± 3.8% at T3 for the conventional group and by 12.1% ± 17.9% at T2% and 13.0% ± 19.9% at T3 for the OD group. The difference between the two groups was not statistically significant at T2 (*p* = 0.359) or T3 (*p* = 0.344).

Implants placed at the premolar/molar area showed an overall higher percentage increase in mean ISQ values at both T2 and T3 compared to implants placed in the incisor/canine area. However, this difference was not statistically significant at T2 (*p* = 0.603) or T3 (*p* = 0.575).

Finally, the area of the maxilla where the implants were placed, along with the insertion torque and implant dimensions, did not appear to influence stability at any of the time points.

### 3.2 Marginal Bone Levels

There was no statistically significant difference between the two different drilling techniques regarding the MBL observed clinically at T2 (Table 3).

**Table 3:** Clinical marginal bone loss.

Clinical marginal bone loss at time of second‐stage surgery (T2) when compared at time of implant placement (T1) (mm)													
Factor	Factor level (*n*)	Mesial			Distal			Buccal			Palatal		
		Mean	(95% CI)	*p*	Mean	(95% CI)	*p*	Mean	(95% CI)	*p*	Mean	(95% CI)	*p*
Treatment	Conventional (19)	0.24	(−0.17, 0.64)	0.632	0.39	(−0.12, 0.91)	0.871	0.47	(0.16, 0.79)	0.988	0.63	(0.23, 1.03)	0.698
	Osseodensification (18)	0.25	(−0.30, 0.80)		0.32	(−0.38, 1.03)		0.50	(−0.05, 1.05)		0.53	(0.04, 1.01)	
Site	Premolars and molars (27)	0.28	(0.16, 0.71)	0.553	0.23	(−0.23, 0.70)	0.689	0.37	(0.00, 0.74)	0.086	0.54	(0.17, 0.90)	0.518
	Incisors and canines (10)	0.15	(−0.19, 0.49)		0.70	(−0.30, 1.70)		0.80	(0.35, 1.25)		0.70	(0.11, 1.29)	
Soft tissue thickness	< 2 mm (3)	−0.17	(‐0.88, 0.55)	0.430	0.67	(−1.23, 2.56)	0.248	0.67	(−0.77, 2.10)	0.531	1.33	(−0.56, 3.23)	0.094
	>= 2 mm (34)	0.28	(−0.07, 0.63)		0.33	(−0.11, 0.78)		0.47	(0.15, 0.79)		0.51	(0.21, 0.82)	
Torque	> 40 (12)	0.71	(−0.15, 1.57)	0.095	0.83	(−0.02, 1.68)	0.108	0.58	(−0.22, 1.38)	0.584	0.83	(0.09, 1.58)	0.529
	< 40 (25)	0.02	(−0.24, 0.28)		0.13	(−0.34, 0.60)		0.44	(0.16, 0.72)		0.46	(0.16, 0.76)	
Implant diameter	3.25 mm (8)	0.50	(−0.27, 1.27)	0.059	0.81	(−0.39, 2.01)	0.238	0.88	(0.29, 1.46)	0.184	1.00	(0.26, 1.74)	0.236
	4.00 mm (23)	0.30	(−0.15, 0.76)		0.40	(−0.07, 0.88)		0.35	(−0.09, 0.78)		0.52	(0.16, 0.76)	
	5.00 mm (6)	−0.33	(−0.60, −0.6)		−0.42	(−1.80, 0.97)		0.50	(0.03, 0.97)		0.25	(−0.84, 1.34)	
Implant length	8.5 mm (13)	0.54	(−0.23, 1.30)	0.479	0.33	(−0.65, 1.31)	0.394	0.58	(0.03, 1.13)	0.893	0.58	(−0.12, 1.27)	0.760
	10.0 mm (20)	0.15	(−0.21, 0.51)		0.50	(0.00, 1.00)		0.45	(0.00, 0.90)		0.65	(0.29, 1.01)	
	11.5 mm (3)	−0.17	(−1.60, 1.27)		−0.33	(−1.05, 0.38)		0.50	(−0.74, 1.74)		0.17	(−1.73, 2.06)	
	13.0 mm (1)	−0.50	N/A		0.00	N/A		0.00	N/A		0.50	N/A	

Radiographically, there was no statistically significant difference between the two different drilling techniques regarding the MBL observed at T2 and T3. Interestingly, there was significantly higher MBL in sites with thin ST sites when compared to thick ST sites both at T2 (*p* = 0.026) and T3 (*p* = 0.017) (Table 4).

**Table 4:** Radiographic marginal bone loss.

Radiographic marginal bone loss (mm)													
		At time of second‐stage surgery (T2) when compared at time of implant placement (T1)						At time of implant restoration (T3) when compared at time of implant placement (T1)					
Factor	Factor level (*n*)	Mesial			Distal			Mesial			Distal		
		Mean	(95% CI)	*p*	Mean	(95% CI)	*p*	Mean	(95% CI)	*p*	Mean	(95% CI)	*p*
Treatment	Conventional (19)	0.12	(−0.05, 0.29)	0.081	0.06	(−0.25, 0.37)	0.391	0.35	(0.14, 0.57)	0.098	0.16	(−0.12, 0.45)	0.391
	Osseodensification (18)	0.54	(0.03, 1.05)		0.43	(−0.09, 0.96)		0.89	(0.36, 1.42)		0.62	(0.09, 1.14)	
Site	Premolars and molars (27)	0.34	(−0.01, 0.69)	0.448	0.26	(−0.09, 0.62)	0.880	0.69	(0.32, 1.06)	0.749	0.34	(−0.03, 0.71)	0.319
	Incisors and canines (10)	0.28	(0.00, 0.56)		0.18	(−0.42, 0.78)		0.40	(0.03, 0.77)		0.50	(0.01, 0.99)	
Soft tissue thickness	< 2 mm (3)	0.27	(−1.33, 1.86)	0.814	1.50	(−0.65, 3.65)	0.026	0.70	(−0.38, 1.78)	0.376	1.40	(0.09, 2.71)	0.017
	≥ 2 mm (34)	0.33	(0.05, 0.61)		0.13	(−0.15, 0.41)		0.61	(0.30, 0.91)		0.29	(0.00, 0.59)	
Torque	> 40 (12)	0.64	(−0.10, 1.39)	0.689	0.53	(−0.26, 1.31)	0.620	0.98	(0.23, 1.73)	0.253	0.68	(−0.08, 1.45)	0.203
	< 40 (25)	0.17	(−0.01, 0.35)		0.10	(−0.16, 0.37)		0.44	(0.20, 0.67)		0.24	(−0.02, 0.50)	
Implant diameter	3.25 mm (8)	0.20	(−0.07, 0.47)	0.741	0.03	(−0.40, 0.45)	0.838	0.35	(−0.04, 0.74)	0.854	0.15	(−0.31, 0.61)	0.539
	4.00 mm (23)	0.40	(0.00, 0.80)		0.27	(−0.15, 0.68)		0.75	(0.31, 1.19)		0.52	(0.08, 0.96)	
	5.00 mm (6)	0.20	(−0.34, 0.74)		0.43	(−0.59, 1.45)		0.45	(0.09, 0.81)		0.17	(−0.36, 0.70)	
Implant length	8.5 mm (13)	0.58	(−0.09, 1.24)	0.911	0.53	(−0.20, 1.26)	0.770	0.75	(0.13, 1.36)	0.251	0.55	(−0.11, 1.20)	0.567
	10.0 mm (20)	0.21	(−0.05, 0.46)		0.08	(−0.22, 0.38)		0.61	(0.26, 0.97)		0.28	(−0.08, 0.64)	
	11.5 mm (3)	0.13	(−0.15, 0.42)		−0.03	(−1.40, 1.33)		−0.07	(−1.19, 1.05)		0.10	(−1.04, 1.24)	
	13.0 mm (1)	0.00	N/A		0.50	N/A		0.90	N/A		1.20	N/A	

## 4 Discussion

The randomized clinical trial herein compared the primary and the short‐term secondary stability of implants placed in sites prepared using implant‐specific CD and OD drills. Preclinical studies have shown promising and encouraging biomechanical and histological results supporting the OD technique. However, it is important to acknowledge that while these studies provide valuable insights, they have several limitations and cannot solely justify the clinical application of the OD technique. The models used in experimental studies often do not anatomically replicate the bone found in the maxilla and/or mandible, particularly as some studies were derived from orthopedic rather than dental literature (Trisi et al. 2016; Huwais and Meyer 2017; Almutairi et al. 2018; Lahens et al. 2016; Lopez et al. 2017; Alifarag et al. 2018; Torroni et al. 2021). Moreover, there was considerable heterogeneity in the methodologies of existing studies, with many having short follow‐up periods (3–6 weeks). Finally, potential conflicts of interest were identified in a number of available studies (Trisi et al. 2016; Huwais and Meyer 2017; Slete et al. 2018).

A randomized split‐mouth control trial by Ibrahim et al. (2020) concluded that OD drilling led to significantly higher primary and secondary implant stability (measured by ISQ) in the posterior maxilla compared to CD (Ibrahim et al. 2020). The study, which only had 10 patients, did not specify the number of operators, and recorded ISQ values only bucco‐lingually, which is not the recommended approach (Ibrahim et al. 2020). In a multicentre trial by Bergamo et al. (2021), results showed that OD provided higher primary and secondary implant stability during the first 6 weeks (Bergamo et al. 2021). Limitations included lack of randomization, multiple implants per patient in both jaws, unreported operator experience, and only a 6‐week follow‐up. Finally, Sultana et al. (2020), in disagreement with the previous studies, found no significant difference between OD and CD in implant stability or MBL. This study had 20 non‐randomized participants, unreported operator details, inclusion of patients with poor oral hygiene, and simultaneous buccal grafting with xenograft (Sultana et al. 2020).

In the current study, at T1, the conventional group had a mean ISQ value of 72.20 ± 2.6 (95%CI, range 65.5–81) while the OD group had a mean ISQ value of 68.1 ± 5.6 (95% CI, range 29–80). ISQ values for the conventional group at T1 agree with the already published normative value for Zimmer Biomet implants which has been reported to have a median value of 73.25 (range: 39–89) at placement, although not all implants in this study were tapered and they were placed in both the maxilla and mandible (Reynolds et al. 2023). Interestingly, the three available clinical trials comparing CD to OD, reported lower ISQ values for CD at T1 (Ibrahim et al. 2020; Bergamo et al. 2021; Sultana et al. 2020). The mean ISQ values for CD at T1 from these studies were reported at 59 ± 17.3 (SD) by Sultana et al. (2020), 59.65 ± 5.39 (SD) by Ibrahim et al. (2020) and 62.00 ± 2.00 (95% CI) by Bergamo et al. (2021). The mean ISQ value for the OD group in the current study, is comparable to that reported by Sultana et al. (2020), which was 66 ± 12.36 (SD) but lower than the values reported by Ibrahim et al. (2020) and Bergamo et al. (2021), which were 74.25 ± 4.95 (SD) and 73.00 ± 2.00 (95% CI), respectively, (Ibrahim et al. 2020; Bergamo et al. 2021; Sultana et al. 2020).

Direct comparison between the results of this study and previous clinical trials is difficult given the heterogeneity in the methodology between the different studies. The study by Bergamo et al. (2021), used three different dental implant systems, only one of which was the same type with the ones used in this current study (Ibrahim et al. 2020). Previous studies have demonstrated that different implant systems have different normative values at placement and following integration (Reynolds et al. 2023; Huwiler et al. 2007; Balleri et al. 2002).

None of the available clinical studies give details regarding how the SmartPeg was tightened to the implants. Given that the literature has shown ISQ values to be influenced by the various techniques SmartPegs are tightened, this could have contributed to the small differences in ISQ values reported between them. A study by Pelegrine et al. (2020), showed that there is a variation of 2.18 ± 1.05 Ncm and 7.51 ± 2.52 Ncm torque amongst different operators, with significantly higher forces shown by males than females (Pelegrine et al. 2020). Additionally, it has been demonstrated that there is a statistically significant difference between ISQ values obtained from hand‐tightening SmartPegs versus tightening SmartPegs at 6 Ncm using a calibrated torque wrench (Naughton et al. 2023). In the current study, all SmartPegs were “finger tightened,” as recommended by Osstell, by the same individual (IP*).

**Figure 4:**
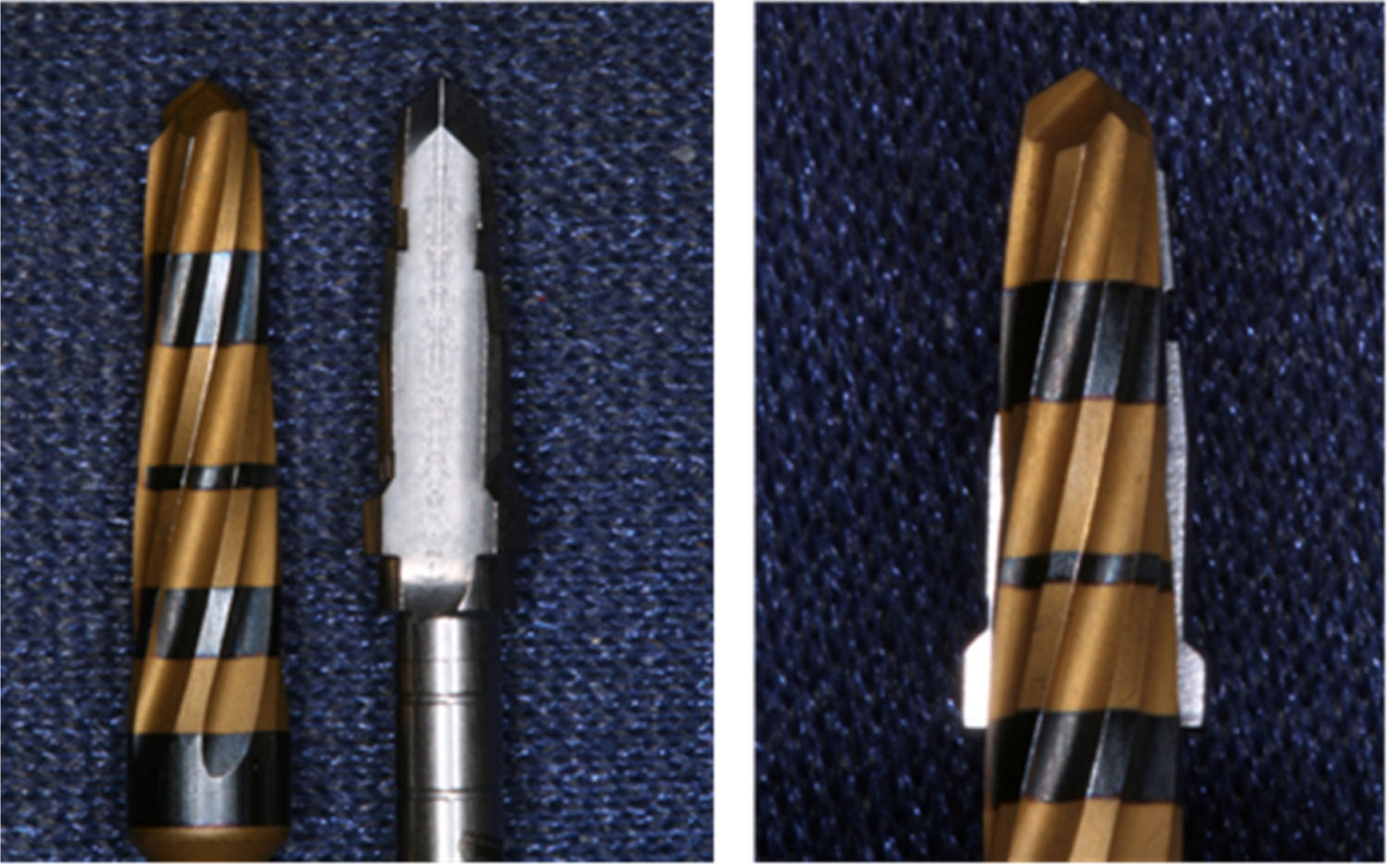
Comparison of the shape of Densah burs with conventional drills specific to BNST ZimVie implants.

During placement of the implant using the OD technique, it became evident that the shape of the Densah burs didn't exactly match the shape of the BNST ZimVie implants (Figure 4). The body of these implants gradually narrows from the neck to the apex, resembling a cone but before the threads start emerging, there is a small step. To accommodate for that, especially in areas with thick cortical bone, the OD protocol had to be adjusted and the coronal 2 mm of the osteotomy widened when necessary to facilitate full sitting of the implant within the osteotomy and keep the mismatch to a low level. The study by Bergamo et al. (2021), which also used the same implant system for some of their sites, did not comment on their methodology and how they dealt with that issue (Bergamo et al. 2021). With the wide variety of implants and their differing anatomical designs available in the market today, it is crucial to consider the compatibility of the drills used with specific implant systems. Furthermore, the design of the ZimVie quad drills, and to this effect the BNST implants themselves, are specifically intended to achieve high primary stability. As a result, it cannot be excluded that comparing more conventional implants might reveal an observable difference in outcomes.

A recent randomized controlled trial compared IT and ISQ values for implants placed in the maxillary premolar area with piezoelectric surgery and OD drills. The piezoelectric device (PD) uses inserts with crescent diameters in a sequence under copious saline irrigation to prepare the osteotomy site using multi‐frequency ultrasonic vibrations. Volution, i‐Res dental implants 4 mm in diameter and 8 or 10 mm in length were used for this study. As neither of the two preparation techniques have been designed for this particular implant system, the level of mismatch between the implant and the osteotomies is unknown. ISQ levels were high for both groups at baseline, for the piezoelectric surgery and 71.3 ± 6.9 for the OD drills. Only piezoelectric group implants, at 3 months after insertion, showed ISQ values higher than at baseline (70.9 ± 4.5 as opposed to 69.3 ± 5.4) (Stacchi et al. 2023).

The percentage change in ISQ values was also calculated in this study. The OD group showed higher percentage increase in ISQ values both at T1–T2 and T2–T3 when compared to the conventional group, but this was not statistically significant. At T1–T2, the OD group showed an increase in ISQ values of 12.1% ± 17.9% (95% CI), while the CD group showed an increase of 4.2% ± 3.7% (95% CI). At T2–T3 ISQ values increased by 13.0% ± 19.9% (95% CI) and 4.0% ± 3.8% (95% CI) for the OD and CD groups, respectively.

Previous studies have shown that there is a marked narrowing of the range of stability values over time in successfully integrated implants (Reynolds et al. 2023). As such, it can be argued that the higher percentage increase in ISQ values for the OD group is because at T1 two patients in that group had much lower ISQ values compared to the mean of the rest of the patients (ISQ: 26.25, 52.25) and hence the higher percentage increase was due to that rather than the effects of OD. After analyzing the results without the two outliers, the ISQ analysis showed that the OD had a similar percentage increase in ISQ values at both T1–T2 and T2–T3 when compared to the CD group (2.05% at T1–T2% and 1.61% at T2–T3).

The correlation between IT and ISQ is not clear in the current literature. In this study, IT did not appear to significantly affect the implant stability at any time point with both low (< 40 Ncm) and high (> 40 Ncm) IT groups showing similar ISQ values. This agrees with the findings of the systematic review by Lages et al. (2018), who found that IT and RFA are independent and have no statistically significant correlation.

Early MBL following implant placement is expected and typically attributed to the surgical trauma and the process of bone remodeling around the implant. There is no agreed value regarding the expected early MBL between implant placement and second‐stage surgery; however, generally studies consider an MBL of 1.5–2 mm in the first year after implant placement as a successful outcome (Galindo‐Moreno et al. 2015; Roos‐Jansåker et al. 2006; Tarnow et al. 2000). In the study herein, marginal bone levels were recorded both clinically and radiographically. None of the differences (or percentage of bone loss) between the two drilling techniques have reached significance. It is important to note that no radiographic stent/jig was utilized to standardize the peri‐apical radiographs; instead, we solely relied on the long cone technique with regular film holders. This should be acknowledged as a limitation of the study.

Finally, the influence of ST thickness, IT, implant site, length and diameter on MBL was also analyzed. The correlation between ST thickness and early MBL in the literature has been conflicting with some studies showing higher early MBL for sites with thin (< 2 mm) ST (Bressan et al. 2023; Berglundh and Lindhe 1996), while others fail to show a correlation (Tomasi et al. 2014; Akcalı et al. 2017). In the current study, ST thickness did not have a statistically significant effect on clinical MBL at T1–T2. However, radiographically, bone under thin ST sites (< 2 mm) showed significantly more marginal loss distally, both at T1–T2 and T2–T3. It is worth noting that this analysis is based on 34 sites with thick ST, while only 3 sites had thin ST.

The sample size for this study was calculated based on the clinical studies available at the time, which reported relatively large mean differences in ISQ between the two drilling techniques. However, our findings revealed only small differences and as a result, a much larger sample size would be indicated.

The main strengths of the study include its robust design as a randomized controlled trial, conducted in adherence to CONSORT guidelines, which enhances its credibility and methodological rigor. Consistency in implant placement and assessment by a single operator and examiner ensures standardization, while comprehensive data collection on ISQ and MBL at multiple time points provides meaningful insights into early healing dynamics. Additionally, in light of the limited number of clinical studies comparing CD and OD, this study adds valuable clinical evidence to a relatively underexplored area, offering a foundation for future research.

The vast majority of the implants placed in the study herein were 4 mm in diameter and 8.5 mm or 10 mm in length but overall, implants of different lengths and diameters were used. Although our statistical analysis was designed to account for these differences, it is possible that they could have partly obscured the actual effect of the drilling techniques.

Other limitations include its small sample size which, despite fulfilling an a priori power calculation, reduces statistical power and limits the generalizability of findings to a broader population. The exclusive focus on maxillary implants and the use of a single implant system (ZimVie) further restricts applicability to other anatomical sites and implant designs. The lack of standardization in radiographic techniques, such as the absence of a radiographic jig, may have affected the accuracy of marginal bone level measurements and the short follow‐up period, focusing only on early healing and secondary stability, leaves long‐term outcomes such as implant survival and peri‐implant health unexamined. Finally, the study's reliance on a single operator enhances standardization but limits generalizability to clinicians with varying expertise levels and the thin soft tissue group's small sample size reduces the reliability of the analysis regarding the influence of soft tissue thickness on MBL.

## 5 Conclusions

As anticipated, there was a notable increase in implant stability over time for both treatment modalities. Moreover, implants inserted into osteotomies made with CD exhibited significantly greater stability compared to those placed using OD drills when this was measured at 3 months (T2). However, the clinical relevance of this difference is questionable, as it was not observed 6 weeks later (T3).

No statistically significant differences were found in the amount of bone loss around implants when comparing OD drills to conventional implant‐specific drills. In general, factors like IT, implant diameter, and length did not show a significant impact on the outcomes assessed in this study. Future research would benefit from larger sample sizes and better standardization of peri‐apical X‐rays at various time intervals. Moreover, considering the diverse anatomical characteristics of dental implants, certain implants may be more suitable for placement using specific Densah burs compared to others.

## Author Contributions

Ioannis Polyzois contributed to the conception and design of the study, contributed to data collection and critically revised the manuscript. Ioanna Politi contributed to the clinical treatment of patients and data collection. Lewis Winning contributed to the conception and design of the study, and critically revised the manuscript. Bahman Honari performed the statistical analysis.

## Conflicts of Interest

The authors declare no conflicts of interest.

## Data Availability

The raw data supporting the conclusions of this article are available by the corresponding author upon reasonable request.
